# Effects of HIF-1 inhibition by chetomin on hypoxia-related transcription and radiosensitivity in HT 1080 human fibrosarcoma cells

**DOI:** 10.1186/1471-2407-7-213

**Published:** 2007-11-13

**Authors:** Adrian Staab, Jürgen Loeffler, Harun M Said, Désirée Diehlmann, Astrid Katzer, Melanie Beyer, Markus Fleischer, Franz Schwab, Kurt Baier, Hermann Einsele, Michael Flentje, Dirk Vordermark

**Affiliations:** 1 Department of Radiation Oncology, University of Würzburg, 97080 Würzburg, Germany; 2 Medical Clinic II, University of Würzburg, 97080 Würzburg, Germany

## Abstract

**Background:**

Hypoxia-inducible factor-1 (HIF-1) overexpression has been linked to tumor progression and poor prognosis. We investigated whether targeting of HIF-1 using chetomin, a disrupter of the interaction of HIF-1 with the transcriptional coactivator p300, influences the radiosensitivity of hypoxic HT 1080 human fibrosarcoma cells.

**Methods:**

Optimal dose of chetomin was determined by EGFP-HRE gene reporter assay in stably transfected HT 1080 cells. Cells were assayed for expression of the hypoxia-inducible genes carbonic anhydrase 9 (CA9) and vascular endothelial growth factor (VEGF) by RT-PCR and for clonogenic survival after irradiation with 2, 5 or 10 Gy, under normoxic or hypoxic (0.1% O_2_, 12 h) conditions in the presence or absence of chetomin (150 nM, 12 h, pre-treatment of 4 h).

**Results:**

Chetomin treatment significantly reduced CA9 and VEGF mRNA expression in hypoxic cells to 44.4 ± 7.2% and 39.6 ± 16.0%, respectively, of untreated hypoxic controls. Chetomin clearly reduced the modified oxygen enhancement ratio (OER') compared to untreated cells, from 2.02 to 1.27, from 1.86 to 1.22 and from 1.49 to 1.06 at the 50%, 37% and 10% clonogenic survival levels, respectively.

**Conclusion:**

HIF-1 inhibition by chetomin effectively reduces hypoxia-dependent transcription and radiosensitizes hypoxic HT 1080 human fibrosarcoma cells *in vitro*.

## Background

Hypoxia in solid tumors is associated with changes in gene expression, mRNA translation and poor prognosis in many tumour entities [1-3]. Furthermore hypoxia enhances radioresistance in tumors and mediates resistance to chemotherapy [4,5]. Hypoxia-inducible factor-1 (HIF-1) is a central regulator of transcriptional response of mammalian cells to oxygen deprivation [6,7].

It has been shown that HIF-1 is associated with poor outcome for multiple cancer types and high HIF-1 expression is a predictor of poor prognosis after radiotherapy [8-11]. Therefore hypoxia inducible factor-1 (HIF-1) has been identified as a potential target to overcome hypoxia induced radioresistance [12].

Kung et al. [13] identified chetomin as a small molecule blocking the HIF pathway. Chetomin disrupts the structure of the CH1 domain of p300, a transcriptional coactivator, thereby precluding its interaction with HIF and attenuating hypoxia-inducible transcription. The aim of the present study was to investigate if HIF-1 inhibition by chetomin affects hypoxia-induced radioresistance in human tumor cells.

## Methods

### Cell culture

Early-passage HT 1080 human fibrosarcoma cells from the American Type Culture Collection (ATCC, Rockville, MD) were maintained under standard conditions as previously described [14]. Cells were cultured in MEM supplemented with 10% fetal bovine serum and 100,000 U/L penicillin and 100 mg/L streptomycin (all from Sigma, St. Louis, MO, USA) with 5% CO_2 _in a well-humidified incubator. For hypoxia experiments, early-passage cells were split and seeded into 80-mm glass Petri dishes at 2 × 10^6 ^cells per dish. On the following day, when cells were still exponentially growing, cells were transferred without further medium change to a Ruskinn (Cincinnati, OH, USA) Invivo2 hypoxic workstation for hypoxia treatment. The oxygen concentration of 0.1% was achieved inside the workstation before transfer of the cells by calibrating the oxygen probe against air according to the manufacturer's instructions; adjusting the instrument settings to the desired O_2 _concentration, 5% CO_2 _and 37°C; and subsequent flooding of the chamber with an appropriate gas mixture of pressurized air, N2, and CO2 through an automated gas mixing module.

Aerobic conditions were maintained in the incubator at 5% CO_2 _and 20% O_2_. Where indicated chetomin (Alexis Biochemicals, San Diego, CA, USA), a pharmacological inhibitor of HIF was used. Different concentrations and (pre-) incubation times of chetomin were assayed in pilot FACS experiments. In RT-PCR and clonogenic survival experiments, chetomin was added in a concentration of 150 nM to fully supplemented medium four hours before treatment with hypoxia. HT 1080 cells were then transferred to the hypoxic workstation (0.1% O_2_, 12 h) or to the well-humidified incubator (12 hours) without changing medium. HT1080 cells were thus treated for 16 hours with chetomin (150 nM) prior to radiation treatment.

### Flow cytometry of 5HRE-hCMVmp-EGFP HT1080 cells

To study the effect of chetomin on hypoxia-responsive element (HRE) binding of HIF-1 and gene transactivation, we used HT 1080 cells stably transfected with enhanced green fluorescent protein (EGFP) under the control of a hypoxia-responsive promoter containing five copies of a hypoxia-responsive element from the human vascular endothelial growth factor (VEGF) gene. HT 1080 cells transfected with the same EGFP under the control of a strong constitutive, hypoxia-independent CMV promoter were used as positive controls as described previously (both cell types kindly provided by J.M. Brown, Stanford University, USA) [14]. The design of the constructs and their application as a reporter system of tumor hypoxia has been previously reported [15].

Cells were exposed to experimental conditions as for PCR experiments in glass Petri dishes. Due to the known requirement of sufficient reoxygenation for the development of hypoxia-dependent, HRE-mediated EGFP fluorescence [14], all samples were returned to aerobic conditions in the incubator for 4 h before FACS analysis as previously described [14]. EGFP fluorescence was measured on a FacsCalibur flow cytometer (Becton Dickinson) as described previously [14]. Dead cells and debris were gated out on the basis of forward and side scatter dot plots.

### RT-PCR

Quantitative real-time RT-PCR assay was performed using the LightCycler instrument and the Fast Start Master Hybridization detection system (both from Roche Diagnostics, Mannheim, Germany) to quantify the mRNA expression of VEGF and CA9. Directly following the hypoxia/chetomin (or respective control) treatment (without reoxygenation), total RNA was purified from 10^6 ^HT 1080 cells using RNeasy Mini kit (Qiagen, Hilden, Germany) according to the manufacturer's instructions.

First-strand cDNA was synthesized from 900 ng total RNA with use of Quantitect Reverse Trancriptase Kit (Qiagen) according to the protocol of the manufacturer. For the amplicon detection, the Fast Start Master Hybridization detection system (Roche Diagnostics) was used taking 0,25 μM (each) of the VEGF- (5'-cctttccctttcctcgaact, 5'-cagaatcatcacgaagtggtgaa) and CA9-specific primers (5'- ttccaatatgaggggtctct 5'-ttcagctgtagccgagagt). Specific probes were either labelled with FAM-TAMRA (6FAM-ctacaccgccctgtgcccac-TMR for quantification of CA9) or LC-Red640 (5'-aagctcatctctcctatgtgctggcct-FL, 5'-LC Red640-ggtgaggtttgatccgcataatctgc-PH, for quantification of VEGF). The following parameters were used: VEGF: 95°C for 600 s: 95°C for 9 s, 55°C for 15 s, 72°C for 20 s and 40°C for 30 s. Forty-five cycles were performed. CA9: 95°C for 600 s: 95°C for 9 s, 60°C for 15 s, 72°C for 20 s and 40°C for 30 s. Forty-five cycles were performed.

### Radiation treatment and clonogenic assay

Immediately after the 12-hour hypoxia treatment or control treatment (± chetomin 150 nM), the glass culture dishes were enclosed in Perspex shells inside the hypoxic workstation and transferred to the linear accelerator. Cells were irradiated with a dose of 2, 5 or 10 Gy (dose rate 2.5 Gy/min, 6 MV photons, room temperature, adequate Perspex bolus). Aerobic cells were irradiated in similar Perspex shells with holes permitting unimpaired air exchange.

Cell survival was quantified by standardized colony-forming assay [16]. HT 1080 cells from untreated or irradiated monolayers were trypsinized and resuspended immediately after treatment, seeded in culture flasks at appropriate dilutions. On day 14, cells were stained with crystal violet, and colonies consisting of at least 50 cells were counted. The plating efficiency (PE) was expressed as the percentage of colonies relative to the number of cells seeded. The ratio of PE of irradiated and respective control cells, correcting for differences in plating efficiency of unirradiated cells, gave the relative survival (S). Survival curves were generated by plotting S as a function of radiation dose (D).

The survival data were fitted with the linear-quadratic model: S(D) = exp [-(αD + βD^2^)] by optimizing variable parameters α and β [17]. A modified oxygen enhancement ratio (OER') was calculated as the ratio of the doses to achieve the same survival at 0.1% O_2 _as at ambient oxygen tensions: OER' = D_hypoxia_/D_normoxia_. OER' was obtained at cell survival levels of 50%, 37% and 10%.

### Statistical analysis

Expression levels of mRNA and clonogenic survival were compared between treatment groups by Mann-Whitney U test using Statistica vs. 6.1 (Statsoft, Tulsa, OK, USA) software (p < 0.05 considered significant).

## Results

### Effects of chetomin on HIF-1-dependent transcriptional activation

**Figure 1:**
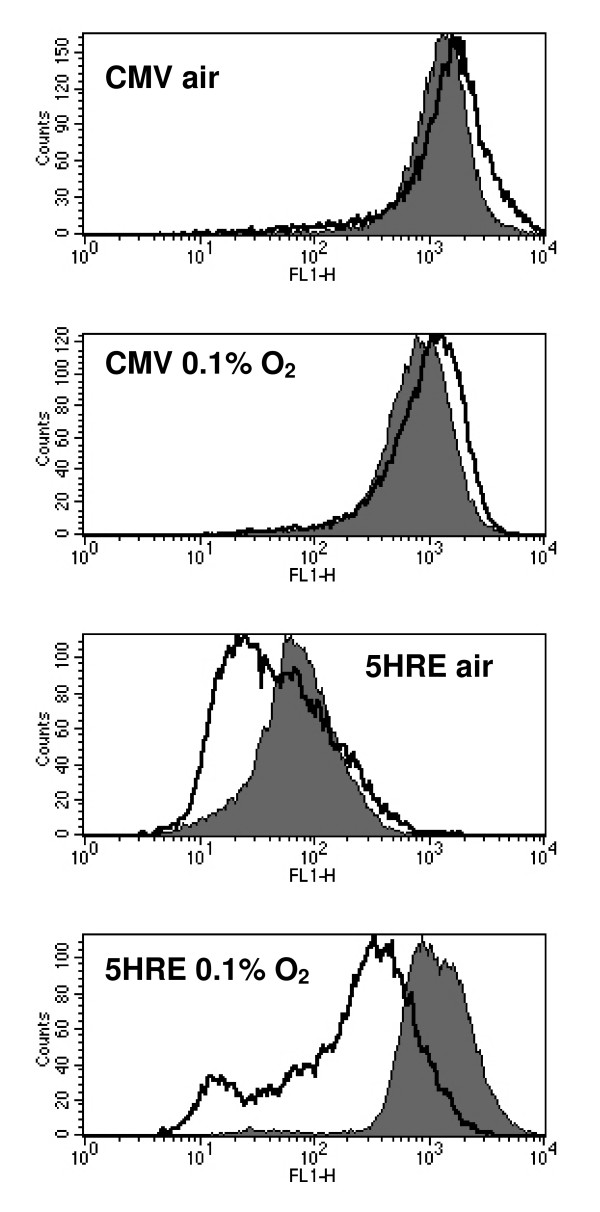
Effect of chetomin on the fluorescence of EGFP under the control of a hypoxia-responsive promoter containing five copies of a hypoxia-responsive element (5HRE) or under the control of a hypoxia-independent constitutive CMV promoter (CMV) in stably transfected HT 1080 human fibrosarcoma cells. Cells were treated in air (20% O_2_) or hypoxia (0.1% O_2_). Black line indicates treatment with chetomin 150 nM, grey shaded area fluorescence of control cells. Chetomin has no effect on EGFP fluorescence in CMV cells, but reduces hypoxia-induced as well as basal normoxic EGFP fluorescence in 5HRE cells.

**Figure 2:**
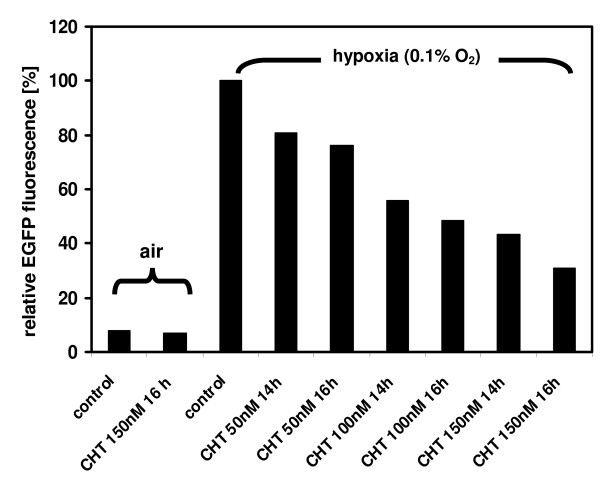
Chetomin (CHT) inhibits the hypoxic activation of hypoxia-responsive-element-(HRE-) mediated EGFP fluorescence in stably transfected HT 1080 human fibrosarcoma cells in a dose and incubation-time dependent manner. After chetomin pre-treatment for two or four hours, HT 1080 cells were cultured for 12 hours under hypoxia resulting in total chetomin treatment times of 14 or 16 h as indicated. A maximum suppression of EGFP fluorescence was achieved when cells were treated with 150 nM chetomin for 16 hours (representative FACS experiment). Higher doses had no additional effect.

In HT 1080 cells stably transfected with a construct of EGFP under the control of a hypoxia-responsive 5HRE-hCMVmp promoter, the addition of chetomin markedly suppressed the hypoxic increase in EGFP fluorescence signal (Figure 1). This suppression of EGFP fluorescence signal was dose and incubation-time dependent (Figure 2). A maximum suppression of EGFP fluorescence signal was measured when chetomin was added four hours prior hypoxia treatment (0.1% O_2_, 12 h) and in a final concentration of 150 nM. Higher doses of chetomin led to an increasing proportion of completely EGFP-negative cells indicating substantial cytotoxicity (data not shown). Chetomin had no effect on HRE-independent EGFP fluorescence as shown by comparable fluorescence in chetomin-treated (150 nM) and control HT 1080 cells transfected with the constitutive CMV promoter (Figure 1).

### Effects of chetomin on VEGF and CA9 mRNA expression

**Figure 3:**
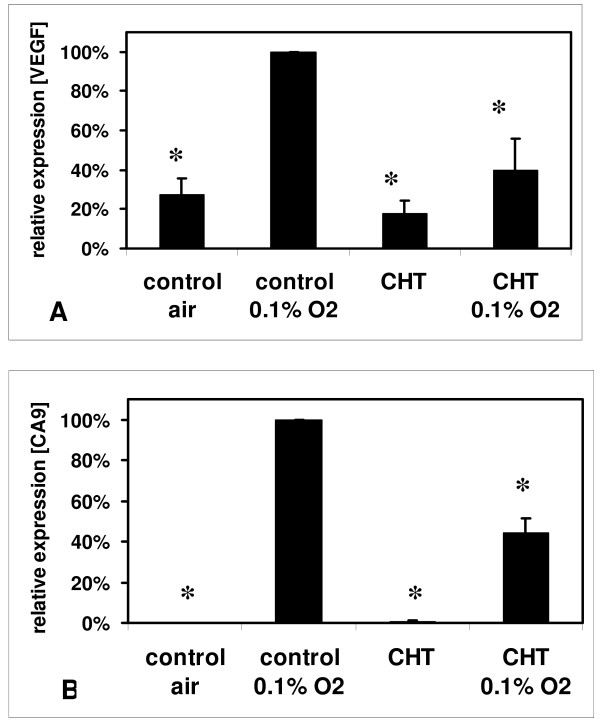
Inhibition of VEGF (A) and CA9 (B) mRNA expression under hypoxia: HT 1080 human fibrosarcoma cells were pre-treated with 150 nM chetomin (CHT) for four hours and transferred for 12 h to hypoxic (0.1% O_2_) or maintained in aerobic (20% O_2_) conditions. Quantitative real-time RT-PCR was performed to quantify the expression of VEGF or CA9 mRNA (n = 3, mean ± sem). Incubation with CHT caused a reduced hypoxic expression of CA9 and VEGF mRNA (* indicates significant difference from hypoxic control, p < 0.05).

Under hypoxic conditions, the treatment of HT 1080 cells with chetomin (150 nM) resulted in a significantly reduced hypoxic VEGF and CA9 mRNA expression (p < 0.05; Figure 3). The mean hypoxic VEGF mRNA expression was reduced to 39.6 ± 16.0% of the hypoxic control (100%) and the mean CA9 mRNA expression was reduced to 44.4 ± 7.2% of the hypoxic control.

### Clonogenic survival

**Figure 4:**
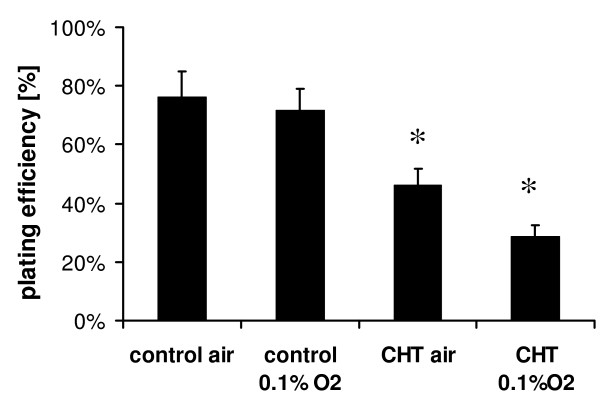
Effects of treatment with *in-vitro *hypoxia (0.1% O2) and/or chetomin 150 nM on the plating efficiency of HT 1080 human fibrosarcoma cells (* indicates significant difference between respective chetomin and control conditions).

Treatment with chetomin (150 nM) alone significantly reduced the plating efficiency under normoxic conditions from 76.2 ± 8.9% (mean ± SEM) to 46.0 ± 5.8% (p < 0.05) and under hypoxic conditions from 71.7 ± 7.3% to 28.6 ± 3.8% (p < 0.05) (Figure 4).

**Figure 5:**
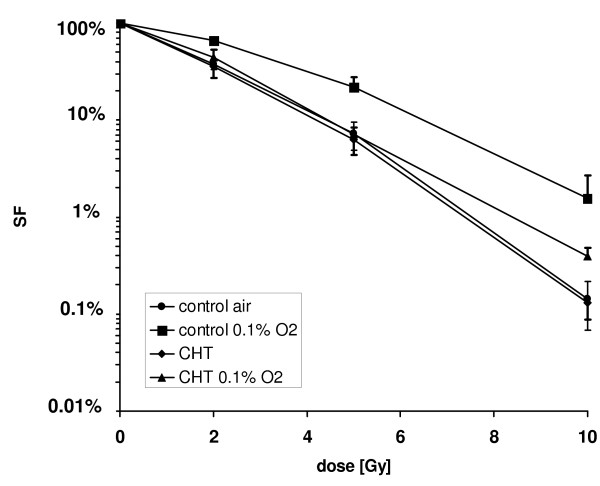
Clonogenic survival of HT1080 human fibrosarcoma cells after radiation treatment in air (20% O_2_) or hypoxia (0.1% O_2_): HT1080 cells were cultured for 12 hours under hypoxia/normoxia and in the presence or absence of 150 nM Chetomin (CHT). Chetomin was added four hours prior to hypoxia (n = 3, mean ± sem). Chetomin 150 nM has no effect on the radiosensitivity of HT 1080 cells in air, but increases radiosensitivity under hypoxic conditions.

Chetomin enhanced radiation treatment efficacy under severely hypoxic conditions (Figure 5). In the absence of chetomin, survival was significantly increased under hypoxic conditions compared to normoxia (p < 0.05). In chetomin-treated cells, survival under hypoxia was significantly increased, compared to normoxia (chetomin) only at the 10 Gy dose level. OER was calculated at 50%, 37% and 10% survival levels obtained from calculated α and β values. Incubation with chetomin reduced the OER' after radiation at all survival levels (Table 1).

**Table 1:** Modified oxygen enhancement ratio (OER')

**Survival**	**OER' (HYP/AIR)**	**OER' (HYP_CHT_/AIR_CHT_)**
10%	1.49	1.06
37%	1.86	1.22
50%	2.02	1.27

Modified oxygen enhancement ratio (OER') derived from clonogenic survival curves after irradiation at oxygen concentrations of 20% (AIR) or 0.1% (Hypoxia = HYP) following treatment with 150 nM chetomin (CHT) where indicated. The modified oxygen enhancement ratio (OER') was calculated as the ratio of the dosis (D) to achieve the same survival as at ambient oxygen tensions: OER' = D_hypoxia_/D_normoxia_. OER was obtained at cell survival of 50%, 37% and 10%.

## Discussion

HIF-1 inhibition has been shown to slow tumor growth in *in-vitro *and *in-vivo *tumor models [13,18] and to act synergistically with other treatment modalities such as radiotherapy [19]. We now show in a human fibrosarcoma cell line that HIF-1 targeting with chetomin (150 nM) suppresses the transcriptional response to hypoxia and reduces hypoxic radioresistance *in vitro*. This is, to our knowledge, the first report of increased radiosensitivity of hypoxic cells *in vitro *in response to chetomin.

Experimental conditions now chosen were based on our previous observation that a near-maximal HIF-1α expression occurs at 12 h of hypoxia at an oxygen concentration of 0.1% O_2_, which represents a level of hypoxia that is frequently observed in solid tumors and radiobiologically relevant [20]. At the dose level of 150 nM, chetomin exhibited a maximum specific effect on HRE-regulated EGFP fluorescence whereas higher doses did not further suppress HRE activation. This dose level was higher than the 50 nM described as effectively suppressing HIF-1 function in Hep 3B cells *in vitro *but lower than the peak tumor concentration in xenograft tumors of 300 nM [13]. In the clonogenic assay, chetomin 150 nM had a cytotoxic effect in aerobic and, in particular, in hypoxic HT 1080 cells. Correcting for this effect, chetomin 150 nM effectively radiosensitized hypoxic HT 1080 cells whereas the radiosensitivity of normoxic cells was unaltered. The effect on normoxic cells could be related to the suppression of basal HIF-1 function which is present in HT 1080 cells under normoxia as suggested by the previously described basal fluorescence of EGFP under control of the hypoxia-responsive promoter [14].

Williams et al. could demonstrate an enhanced response to radiotherapy in tumours deficient in the function of hypoxia-inducible factor-1 [21]. HIF-1α antisense treatment suppressed HIF-1α expression by up to 80% under both normoxic and hypoxic conditions and resulted in significant resistance of U87 malignant glioma cells to the cytotoxicity of cisplatin, etoposide, and vincristine [22]. Sasabe et al. demonstrated that down-regulation of HIF-1α expression by small interfering RNA enhances the susceptibility of oral squamous cell carcinoma cells to chemo- and radiotherapy [23]. Pore et al. [24] showed that decreases of both VEGF and HIF-1α expression result in radiosensitization *in vivo *and *in vitro*.

In contrast to results of the present study, Arvold et al. [25] showed that in mouse embryonic fibroblasts hypoxia-mediated radiation resistance is independent of HIF-1α. Furthermore, Moeller at al. detected that radiation activates HIF through reoxygenation-induced stabilization of HIF-1 dimer through free radical intermediates and reoxygenation-mediated depolymerization of hypoxia-induced translational suppressors known as stress granules [26].

Published results and our data suggest that HIF is an attractive target to overcome hypoxia-induced radioresistence. Kung et al. [13] could demonstrate *in vitro *and *in vivo *that administration of chetomin inhibited hypoxia-inducible transcription within tumors and inhibited tumor growth, thereby establishing the feasibility of disrupting a signal transduction pathway by targeting the function of a transcriptional coactivator with a small molecule *in vivo*. In the present study, mechanistic experiments focussing on the mode of HIF-1 inhibition by chetomin, in particular its interaction with p300, as presented by Kung et al. [13] were not performed. The findings now presented are limited to one *in-vitro *model and one HIF-1 targeting agent. The selection of clinically promising HIF-1 targeting agents will require a broader screen of several substances in a panel of cell lines *in vitro *with confirmation of results in experimental *in-vivo *models. Recent studies suggest that the success of HIF-1 inhibition *in vivo *may critically depend on other factors of the tumor microenvironment such as glucose metabolism and pH [27,28] and on the ability of any given strategy to eliminate virtually all HIF-1 function as cell mixing experiments described by Williams et al. [21] indicated that 1% of cells with intact HIF-1 function within a tumor are sufficient to preserve tumor growth.

In conclusion, we could demonstrate that HIF-1 inhibition with chetomin downregulates the expression of HIF-1-regulated genes (CA9, VEGF) and enhances radiation response under severely hypoxic conditions in human HT 1080 cells. Further studies must show whether selective inhibition of HIF-1 will increase radiosensitivity *in vivo*.

## Competing interests

The author(s) declare that they have no competing interests.

## Authors' contributions

AS designed the study, performed experimental procedures, analyzed the data and drafted the manuscript.

JL participated in study design, performed experimental procedures, analyzed the data and reviewed the manuscript.

HMS participated in study design and reviewed the manuscript.

DD performed experimental procedures, analyzed the data and reviewed the manuscript.

MB performed experimental procedures, analyzed the data and reviewed the manuscript.

MaF performed experimental procedures, analyzed the data and reviewed the manuscript.

FS aided in experimental procedures and reviewed the manuscript.

KB analyzed the data and reviewed the manuscript.

HE aided in study design and reviewed the manuscript.

MiF aided in study design and reviewed the manuscript.

DV designed the study, performed experimental procedures, analyzed the data and drafted the manuscript.

All authors approved the final version of the manuscript.

## Pre-publication history

The pre-publication history for this paper can be accessed here:


